# Semi-automated quantitative Drosophila wings measurements

**DOI:** 10.1186/s12859-017-1720-y

**Published:** 2017-06-28

**Authors:** Sheng Yang Michael Loh, Yoshitaka Ogawa, Sara Kawana, Koichiro Tamura, Hwee Kuan Lee

**Affiliations:** 1 Imaging Informatics Division, Bioinformatics Institute, 30 Biopolis Street, 07-01, Matrix, Singapore, Singapore, 138671 Singapore; 2 Department of Biological Sciences, Tokyo Metropolitan University, Hachioji, Tokyo, 192-0397 Japan; 3 Research Center for Genomics and Bioinformatics, Tokyo Metropolitan University, Hachioji, Tokyo, 192-0397 Japan

**Keywords:** *Drosophila*, Image processing, Wing morphometrics, Automated detection

## Abstract

**Background:**

*Drosophila melanogaster* is an important organism used in many fields of biological research such as genetics and developmental biology. *Drosophila* wings have been widely used to study the genetics of development, morphometrics and evolution. Therefore there is much interest in quantifying wing structures of *Drosophila*. Advancement in technology has increased the ease in which images of *Drosophila* can be acquired. However such studies have been limited by the slow and tedious process of acquiring phenotypic data.

**Results:**

We have developed a system that automatically detects and measures key points and vein segments on a *Drosophila* wing. Key points are detected by performing image transformations and template matching on *Drosophila* wing images while vein segments are detected using an Active Contour algorithm.

The accuracy of our key point detection was compared against key point annotations of users. We also performed key point detection using different training data sets of *Drosophila* wing images. We compared our software with an existing automated image analysis system for *Drosophila* wings and showed that our system performs better than the state of the art. Vein segments were manually measured and compared against the measurements obtained from our system.

**Conclusion:**

Our system was able to detect specific key points and vein segments from *Drosophila* wing images with high accuracy.

**Electronic supplementary material:**

The online version of this article (doi:10.1186/s12859-017-1720-y) contains supplementary material, which is available to authorized users.

## Background

Recent advances in high-throughput sequencing technology have enabled us to obtain genome information from any kind of organism [1]. For humans, more than a thousand of genome sequences and single nucleotide polymorphisms among them have been characterized [2]. This further motivated genome-wide association studies (GWAS) in the literature with the hopes of discovering the variations responsible for genetic diseases and traits in the genome sequences. The strategy of investigating the relationship between genotype and phenotype should succeed in discovering the genetic basis for any inherited traits in any kinds of organisms. However, in many higher animals other than human beings, the description of phenotype needs manpower with expertise in morphology of the animals under consideration; such a dependency of this approach on available manpower may be a critical bottleneck of GWAS. Hence, development of an automated system for describing phenotype may be one of the best solutions to this problem.

Fruit flies, in particular *Drosophila melanogaster* is an important organism used for biological research fields particularly in genetics and developmental biology. Moreover, the complete genome sequences of this species and its related species [3] opened up an ideal opportunity for comparative genomics analyses for the systematic understanding of phenotype and genotype relationships. Species belonging to the genus *Drosophila* are particularly useful for this purpose because there are more than 2000 described species in the genus *Drosophila* [4], each of which has distinct phenotypic characters. On the other hand, this situation makes it difficult for ordinary *Drosophila* researchers to distinguish morphological differences among different species and only a limited number of researchers who are experts in *Drosophila* taxonomy can recognize key morphological characters to identify the species.

The use of wing morphometrics for species identification have been demonstrated on a variety of insect species [5–9], because of the simple two dimensional structures with clear-cut patterns of veins and their crossings, which are frequently utilized as landmarks to measure distances and angles among them. There is much interest in quantifying wing structures of *Drosophila* [10–14] and *Drosophila* wings have been widely used to study the genetics of development, morphometrics and evolution [15–20]. Advances in technology have increased the ease in which images of insect wings can be acquired. However the extraction of phenotypic information for further research and analysis is a manual and time consuming process. This has motivated the creation of several software which aims to automate this task. One common method involves using software for easy annotation of landmarks on the wing (http://life.bio.sunysb.edu/ee/rohlf/software.html, http://www.hockerley.plus.com/). However annotating landmarks is time consuming and prone to errors [21]. More advanced software attempt to find the location of landmarks and vein lengths using a variety of image processing and optimization methods. These methods tend to involve fitting a set of bezier splines onto the wing veins. WINGMACHINE [22] is a program that automatically detects and measures the positions of veins and edges of wing blades from live flies. MorphoJ [23] performs geometric morphometrics analysis on morphological landmark inputs. Although MorphoJ does not detect and extract landmarks, it is a useful tool for studying data on shape combined with molecular genetics or ecological information. Automated systems to detect and extract information from wings from a variety of other insect species exist [24–28]. However only a few of these programs are able to extract specific unique key points that are used for the prediction of the fly species. Crnojevic et al. [28] trained a svm classifier based on Histogram of Oriented Gradient (HOG) and Complete Local Binary Pattern (CLBP) features for vein junction detection of hoverflies. In addition the junctions were used to construct convex hulls which were used to discriminate 4 different hoverfly species. Thus a more specific and customized software is needed to automate the detection and prediction process of *Drosophila* wings. As mentioned previously, the wing morphology of *Drosophila* species is highly favorable for automated image analysis [22]. Therefore, in this paper we introduce an automated system that locates specific keypoints on a *Drosophila* wing image and performs morphometrics measurement of several important vein segments. The extraction of these phenotypic data serves as a base for future studies and research of *Drosophila* wings such as specie prediction.

The software requires the user to annotate three specific key points on the fly wing before key points and vein segments are calculated automatically. The program provides an intuitive user interface and users can manually edit key points and vein segment lengths if the prediction is inaccurate. In this contribution, we report the performance of our program and perform a comparison against WINGMACHINE [22] an existing program that automates the measurement of *Drosophila* wings.

## Implementation

### Data set

A total of 959 flies from 16 Drosophila species was collected in a wood on the Minami-osawa campus of Tokyo Metropolitan University in Tokyo, Japan. The species identified according to their morphology were *Dichaetophora acutissima*, *Drosophila annulipes*, *D. bizonata*, *D. busckii*, *D. curviceps*, *D. hydei*, *D. immigrans*, *D. lutescens*, *D. rufa*, *D. sternopleuralis*, *D. suzukii*, *Hirtodrosophila sexvittata*, *Liodrosophila aerea*, *Microdrosophila sp.*, *Scaptodrosophila coracina* and *Scaptomyza graminum* (Table 1). A one-sided wing was taken from each individual and placed in 8 *μ*l mounting solution (ethanol : glycerol = 2 : 3) dropped on a glass slide. After a coverslip was placed, the slide was placed under a stereomicroscope (Nikon SMZ-10A) and the wing photograph was taken by a digital camera (Canon EOS 40D), where the original image resolutions was 1936 ×1288, 2816 ×1880, or 3888 ×2592 pixels.

**Table 1:** Species and the number of samples used in this study

Species	Female	Male
*Dichaetophora acutissima*	20	25
*Drosophila annulipes*	31	27
*Drosophila bizonata*	31	31
*Drosophila busckii*	35	41
*Drosophila curviceps*	20	26
*Drosophila hydei*	11	20
*Drosophila immigrans*	41	41
*Drosophila lutescens*	14	42
*Drosophila rufa*	41	41
*Drosophila sternopleuralis*	42	42
*Drosophila suzukii*	22	19
*Hirtodrosophila sexvittata*	41	41
*Liodrosophila aerea*	41	41
*Microdrosophila sp.*	15	10
*Scaptodrosophila coracina*	5	34
*Scaptomyza graminum*	36	32

**Fig. 1:**
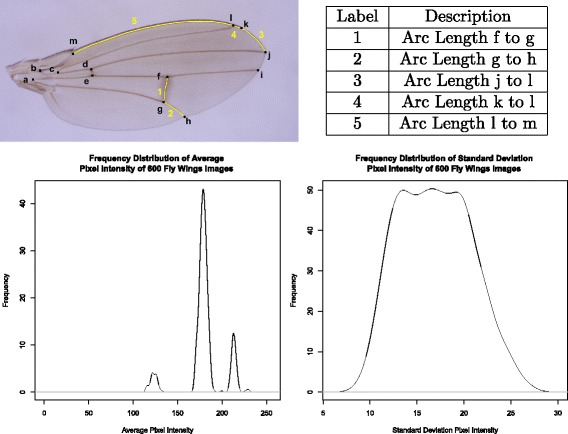
Figure shows the thirteen keypoints and vein segments that can be automatically measured by the software described in this paper. In additional, the mean and standard deviation of the pixel intensities of all 600 images used are shown as well

A total of 600 images were used for image processing. Figure 1 shows the arc-lengths to be measured as well as the mean and standard deviation of the pixel intensities of all 600 images. To conserve memory usage and improve processing speed, small image patches around the manually annotated key points were cropped and stored in small image patch files. In practise, only image patches near the key point locations were needed for key point detection. All images are also converted to gray scale images. Let annotated images be denoted by *D*_*m*_, where *m*=1⋯*N* and *N*=600 is the total number of images.

### Key point detection

To identify a species from the wing image 13 key points are needed. The key points are labelled ‘a’ to ‘m’ and can be found in Fig. 1. Key point ‘k’ is the boundary of the costal fringe and the others are intersections of veins. Given a new, unannotated fly wing image *I*, key points detection is carried out in two steps. The first step aligns the annotated images *D*_*m*_ and the input image *I*. In the next step, key points are detected using template matching.

**Fig. 2:**
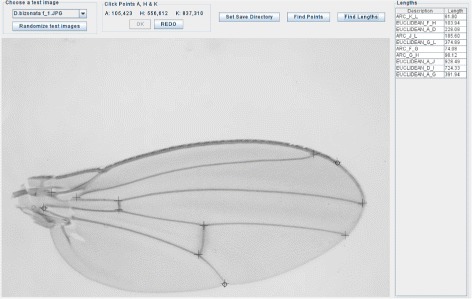
Screen shot of the user interface

**Fig. 3:**
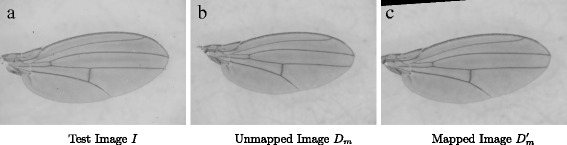
**a** shows an example input image *I* while **b** shows an unmapped image *D*_*m*_. After applying a transformation matrix to *D*_*m*_, the mapped image $D^{\prime }_{m}$ is obtained (**c**)

To align the input image *I* with *D*_*m*_, the user is required to click the location of the three key points ‘a’, ‘h’ and ‘k’ on *I* (see Fig. 1). A screen shot of the software can be found in Fig. 2. An affine transformation matrix *Q*_*m*_ is then calculated by matching the key points ‘a’, ‘h’ and ‘k’ in *I* and *D*_*m*_. Hence, each image in the data set *D*_*m*_ is associated with one affine transformation *Q*_*m*_, *m*=1,⋯*N*. This transformation matrix is then used to transform all images in the data set from *D*_*m*_ to $D^{\prime }_{m}$. Figure 3 shows an example of transforming *D*_*m*_ to $D^{\prime }_{m}$. In our implementation, only pixels within a small image patch near the annotated key points of *D*_*m*_ are transformed. This process greatly increase the processing speed. As an affine transformation is used to align the images, the software is capable of handling fly wings of any orientation.

#### Template matching

Image patches between *I* and *D*_*m*_ are matched and the best match locations are predicted to be the key point locations. Define *w* ^*I*^(*x,y*,*θ*) to be an image patch centred at (*x,y*), oriented at an angle *θ* extracted from the image *I*. Similarly, let $w^{D^{\prime }_{m}}(x,y,\theta)$ be an image patch extracted from the image $D^{\prime }_{m}$. The objective is to find the best match between *w* ^*I*^ and $w^{D^{\prime }_{m}}$ by adjusting the image patch parameters *m,x,y*,*θ*.

**Fig. 4:**
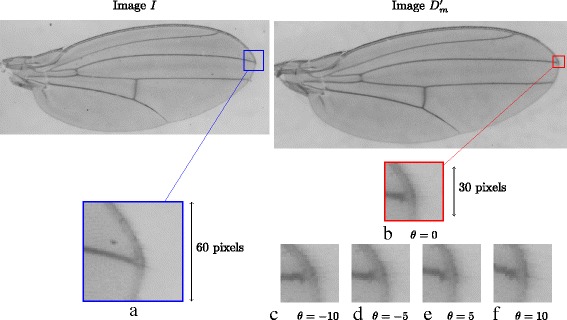
Figure showing the target location and templates created using $D^{\prime }_{m}$ for key point *j*. The target location $w^{D^{\prime }_{m}}\left (x^{\prime {m}}_{j},y^{\prime {m}}_{j},\theta =0\right)$ (**a**) is created at the location $\left (x^{\prime {m}}_{j},y^{\prime {m}}_{j}\right)$ on image *I*. **c**, **d**, **b**, **e** and **f** shows the 5 templates $w^{D^{\prime }_{m}}\left (x^{\prime {m}}_{j},y^{\prime {m}}_{j},\theta \right)$ created at $\left (x^{\prime {m}}_{j},y^{\prime {m}}_{j}\right)$ on image $D^{\prime }_{m}$ with *θ*=−10,−5,0, 5, 10 degrees respectively

We shall describe the template matching procedure using key point *j* as illustration. The procedure for matching other key points is similar. The location of key point *j* in *D*_*m*_, $x^{m}_{j},y^{m}_{j}$ is known since *D*_*m*_ has been manually annotated. The transformed location $x^{\prime {m}}_{j},y^{\prime {m}}_{j}$ can be calculated using *Q*_*m*_. Five templates $w^{D^{\prime }_{m}}\left (x^{\prime {m}}_{j},y^{\prime {m}}_{j},\theta \right)$ are constructed with *θ*=−10,−5,0,5,10 degrees. Fig. 4 c, d, b, e and f show examples of these five templates. Next, we want to match these templates to a target location $w^{I}\left (x^{\prime {m}}_{j},y^{\prime {m}}_{j},0\right)$ in image *I*. One last step before the matching process is to normalize the pixel intensities within the patches by performing a linear transformation to make the range of pixel intensities within the image patch between 0 and 255.

**Fig. 5:**
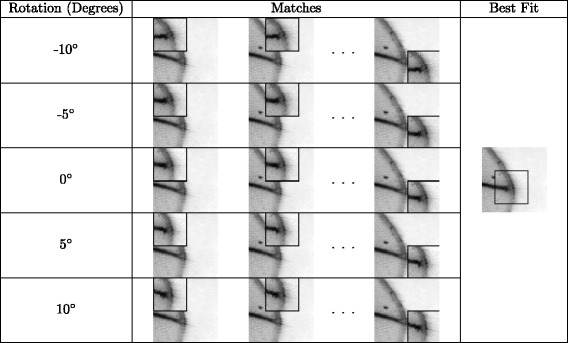
The process of template matching is shown here. Each patch is moved across the search area. The Normalized Squared Summed of Pixel Intensity Differences is calculated at each position. The position with the least pixel-wise difference corresponds to the key point location

Matching is done by sliding the window $\tilde {w}^{D^{\prime }_{m}}\!\left (\!x^{\prime {m}}_{j},y^{\prime {m}}_{j},\theta \!\right)$ with respect to $\tilde {w}^{I}\left (x^{\prime {m}}_{j},y^{\prime {m}}_{j},0\right)$. $\tilde {w}^{D^{\prime }_{m}}\left (x^{\prime {m}}_{j},y^{\prime {m}}_{j},\theta \right)$ and $\tilde {w}^{I}\left (\!x^{\prime {m}}_{j},y^{\prime {m}}_{j},0\right)$ are the normalized image patches. Figure 5 illustrates the matching process. The matching score is given by,

1 $$ s_{m,\theta}(c_{x},c_{y}) = \left\| {\tilde{w}}^{{D}^{\prime}_{m}}\left(x^{\prime}{m}_{j},y^{\prime}{m}_{j},\theta\right) - \tilde{w}^{I}\left(x^{{\prime}^{m}}_{j}-c_{x},y^{\prime}{m}_{j}-c_{y},0\right) \right\|  $$

*c*_*x*_,*c*_*y*_ is the shift of the center locations between the image patches, with −15≤*c*_*x*_,*c*_*y*_≤15. ∥·∥ is the Euclidean norm. Finally, the predicted key point location corresponds to the best match among all shifts, orientations and template images *D*_*m*_.

2 $$ \left(c_{x}^{*},c_{y}^{*},\theta^{*},m^{*}\right) = \arg \min s_{m,\theta}(c_{x},c_{y})  $$

and the key point *j*’s coordinate is,

3 $$ \left(x^{I}_{j},y^{I}_{j}\right) = \left(x^{{\prime}^{m^{*}}}_{j} - c_{x}^{*}, y^{{\prime}^{m^{*}}_{j}} - c_{y}^{*}\right)  $$

The above process is repeated for the remaining key points on *I*. The details of the above algorithm can be found in Algorithm 1 KEYPOINT-DETECTION in Additional file 1.

### Arc length calculations

Tracing the arcs as depicted in Fig. 1 is done using active contours while the Euclidean distances between key points are calculated trivially. In the active contour method, an estimate of the desired curve is first generated using a template. The curve is then evolved using a variational principle via a gradient descend method. Two essential ingredients for applying the active contour method are estimation of the initial curve and the existent of a good gradient to guide the curve towards the desired position.

#### Initialization of active contours

The initialization of active contour curves is critical for finding the fly wing vein accurately. If the initial curve lies far away from the vein, active contour might evolve this arc to another vein.

For example, setting the initial curves to straight lines works well for arcs fg, gh, jl and kl as these arcs are relatively straight. This is not the case for arc ml as there is another vein just below it. Active contour will tend to evolve this curve towards the vein below ml.

A good initialization of curves can be obtained by mapping veins of a template fly wing image to the input image *I*. This template wing should provide a good representation of wings for all different fly wing species. This mapping is done by aligning the template fly wing image with the input image. The alignment process has been described in the key point detection section.

**Fig. 6:**
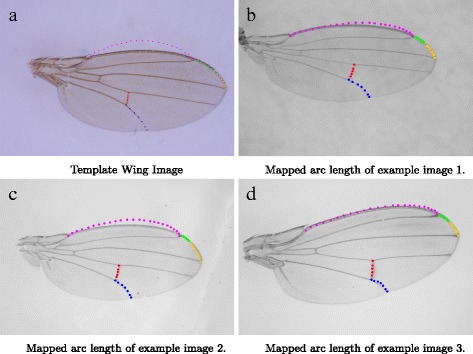
**a** shows a wing from a male *Drosophila rufa* fly which is being used as a template wing. This image is chosen as its key point k is equidistant to both key point j and l. **b** - **d** shows the mapped arc lengths using the template wing for 3 different images. The colours *red, blue, orange, green* and *magenta* represents the arc segments fg, gh, jl, kl, lm respectively

Figure 6 illustrates this concept. First a fly wing image is chosen to be the template wing image (Fig. 6 a). We have selected our template wing such that key point k is equidistant to both key point j and l. Mapped arcs will be warped badly if key point k is not equidistant to both key points j and l. Different arcs are drawn using different colours in Fig. 6 for clarity. Arc ml is drawn outside of the template wing due to two reasons. The first is that the arc ml of the template wing that we have chosen is quite flat and does not provide a suitable representation of other fly wings’ ml arcs, which have more curvature. Secondly, it can be noticed that there is another vein just below arc ml that lies close to arc ml. Active Contour might evolve the mapped arc to the wrong vein if the mapped arc happens to lie closer to that vein and not arc ml. To prevent this, the arc ml is drawn outside the template wing image so that the mapped arc also lies outside the fly wing. Some results of this mapping are shown in Fig. 6 b – d. One can now notice how the mapped arc ml of Fig. 6 c lies outside the wing, rather than in between the two veins.

#### Generation of gradients for active contour optimization

**Fig. 7:**
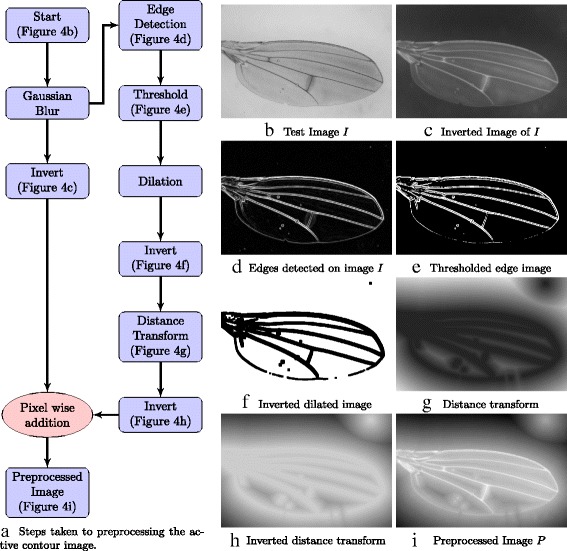
Figure shows the preprocessing of image *I* (**b**) before the arc lengths are found. **c** and **h** shows the inverted image of *I* and the inverted distance map of *I* respectively. Both images are added pixel-wise to get *P*, the processed image (**i**)

Figure 7 shows how the input image is processed to produce a gradient for active contour optimization. The main idea is to make the veins’ pixels very bright and for them to be surrounded by pixels that become darker as they become further away from the veins. A step-by-step work flow of how the preprocessed image is obtained is shown in Fig. 7 a. The original image (Fig. 7 b) is blurred and inverted to obtain an image where the veins’ pixels are very bright. The result is shown in Fig. 7 c. A second image is needed to help produce a smooth downwards gradient away from the vein. Edges (Fig. 7 d) are found on the blurred image before applying a threshold to obtain a binary image (Fig. 7 e). The binary image is dilated to make the fly wing veins thicker before inverting the image (Fig. 7 f). A distance transform (Fig. 7 g) is applied before inverting the image one last time (Fig. 7 h). The two images (Fig. 7 c and h) are then added pixel-wise to produce the preprocessed image *P* (Fig. 7 i). This process is integrated in the software and is done automatically before performing Active Contour.

#### Active contour formalism

Given an image *P*, the arc length between 2 key points can be found by finding the path that follows high intensity pixels between the two points. Curves are also represented by connecting short straight line segments. Given 2 key points *p*_*a*_=(*x*_*a*_,*y*_*a*_) and *p*_*b*_=(*x*_*b*_,*y*_*b*_), *n* straight line segments are created to connect *p*_*a*_ to *p*_*b*_ by *n*−1 intermediate points (*x*_1_,*y*_1_)…(*x*_*n*−1_,*y*_*n*−1_) where (*x*_0_,*y*_0_)=(*x*_*a*_,*y*_*a*_) and (*x*_*n*_,*y*_*n*_)=(*x*_*b*_,*y*_*b*_). The *ith* line segment thus has a length of $\sqrt {(x_{i}-x_{i-1})^{2} + (y_{i}-y_{i-1})^{2}}$. An objective function for the active contour can then be defined as,

4 $$ {}L(x_{1},y_{1},{\ldots}x_{n-1},y_{n-1}) = f(x_{0},{\ldots}y_{n},P) + \alpha\sum^{n}_{i=1}(l_{i}-l_{0})^{2}  $$

where *l*_0_=∥*p*_*a*_−*p*_*b*_∥/*n* is a constant and *α*≥0 is a tuning parameter. *l*_0_ is the equilibrium length of the line segments, i.e. if *l*_*i*_=*l*_0_ then its contribution to the second term in Eq. 4 is zero. The function *f* is chosen such that *f* is small along paths of high image intensity. To make the active contours invariant with respect to fluctuations of the overall image intensity, we make *f* to be invariant to image intensity multiplication, i.e. *f*(*x*_0_,…*y*_*n*_,*P*)=*f*(*x*_0_,…*y*_*n*_,*aP*). Hence *f* can be defined as:

5 $$ {\begin{aligned} f(x_{0},{\ldots}y_{n},P) = \frac{\langle P \rangle}{2} \sum^{n}_{i=1} l_{i} \left[\frac{1}{P(x_{i-1},y_{i-1})+\epsilon} + \frac{1}{P(x_{i},y_{i})+\epsilon}\right] \end{aligned}}  $$

6 $$ \langle P \rangle = \frac{1}{2}\left[P(x_{0},y_{0}) + P(x_{n},y_{n})\right]  $$

〈*P*〉 is the normalization on the image intensity such that *f* becomes invariant under image intensity multiplication. 0<*ε*≪1 is a small regularizer to prevent numerical overflow. The desired curve can be found by minimizing the objective function:

7 $$ L\left(x^{*}_{1},y^{*}_{1},{\ldots}x^{*}_{n-1},y^{*}_{n-1}\right) = \min_{x_{1},{\ldots}y_{n-1}} L(x_{1},{\ldots}y_{n-1})  $$

using gradient descend method,

8 $$ \left(x_{k}^{(t+1)},y_{k}^{(t+1)}\right) = \left(x_{k}^{(t)},y_{k}^{(t)}\right) - \eta\partial_{k}L\left(x_{1}^{(t)},{\ldots}y_{n-1}^{(t)}\right)  $$

Finally, the arc length can then be calculated by:

9 $$ l_{a,b} = \sum^{n}_{i=1} \sqrt{\left(x^{*}_{i}-x^{*}_{i-1}\right)^{2} + \left(y^{*}_{i}-y^{*}_{i-1}\right)^{2}}  $$

The details of the algorithm can be found in Algorithm 2 ARC-LENGTH in Additional file 1.

### Species identification from fly wing images

The *Drosophila* species can be identified from the wing image using 13 key points (Fig. 1). The centroid of wing was estimated as the centroid of the octagon whose vertices were the key points ‘a’, ‘h’, ‘i’, ‘j’, ‘k’, ‘l’, ‘m’ and ‘b’. The centroid of the octagon was computed from the area-weighted average of the centroids of the five triangles split from the octagon. Then, Euclidean distances from the wing centroid to the 13 key points were measured and normalized by dividing by the distance between ‘a’ and ‘j’ (wing length).

In addition to the normalized distances from 13 key points to the centroid, we measured three traditional wing indices, costal-index, c3 fringe and 5x-index [29–31] to identify the species from a wing image. The costal-index is defined as the ratio of the length of the vein l-m to that of the vein j-l. The C3 fringe is the ratio of the length of the vein k-l to that of the vein j-l. The 5X-index is the ratio of the length of the vein g-h to that of the vein f-g. Because arc lengths of wing veins were required to compute these indices, we used a fitting of veins to Bezier paths. As a result, for each wing, we obtained 16 variables to construct the training dataset and measured their values for 589 flies from 15 species excluding *Microdrosophila sp.*, which was used as a sample for a negative control as will be explained later.

With this size of the training dataset, however, utilization of all of the 16 variables did not show the best performance in the species identification in our preliminary test, where the data from 588 files were used to construct the training dataset and the remaining data from one fly was used as the test data. Therefore, we examined the conditions that may give the best result among all the possible numbers (2-16) and possible combinations of these numbers of variables (65519 patterns in total) and found that the best result was obtained when six variables, the distances from the key points e, f, g and m to the centroid, costal-index and c3-fringe, were employed. Accordingly, we used only these six variables for the following discriminant analysis.

The mean vector and the variance and covariance matrix of the six variables were computed for the 15 species and for male and female separately excluding *Drosophila hydei* female and *Scaptodrosophila coracina* female due to a small sample size. Therefore, we obtained the vectors for 28 groups in total. Then, the squared Mahalanobis’ distance, D^2^, [32] from each wing to each group was computed. Following De Maesschalck et al. ([33]), we used $D_{i,j}^{2}=(x_{j}-\hat {\mu _{i}})^{T}\hat {S_{i}}^{-1}(x_{j}-\hat {\mu _{i}})$, where *x*_*j*_ denotes the vector of *j*-th wing, $\hat {\mu _{i}}$ and $\hat {S_{i}}$ denote the mean vector and the variance and covariance matrix for *i*-th group, with the aid of the R package ([34]).

For each wing image from unknown sample, the *D* ^2^ values were computed with $\hat {\mu _{i}}$ and $\hat {S_{i}}$ for the 28 groups and the group that gave the smallest *D* ^2^ value was identified to be the species of the sample. The statistical significance of the goodness of fit to the inferred group was obtained by the chi-square test with *n* degrees of freedom. When the smallest *D* ^2^ value was larger than the expected value for the best-fit species at the 0.1% level, the sample’s species was identified as ‘unknown’.

To examine the efficiency of species identification by the discriminant analysis, we used 370 additional wing images not included in the training data from 15 species including *Microdrosophila sp.*, which were absent in the training data and thus expected to be identified as ‘unknown’ species if the method worked correctly.

### Evaluation metric of key point detection

The key points of each fly wing in the data set is found using the methods described above. To test the accuracy of the key point detection algorithm, 15 fly wing images, one from each species, is manually annotated by 10 users. This give us 10 X,Y coordinates for each key point for each of the 15 fly wing images. The covariance matrix, which represents the spread of the 10 annotated points for a single key point, can be obtained from the manually annotated data. These covariances matrices are then used for benchmarking the accuracy of the automated methods.

A total of 15 covariance matrices, 1 for each annotated image, are obtained for a single key point. The 15 covariance matrices are summed and averaged to obtain the final covariance matrix for a single key point. This process is done for all key points. Table 2 shows the covariance matrix obtained for each key point. One might notice that the covariance matrix of key points ‘a’, ‘b’, ‘c’, ‘k’ and ‘m’ contains larger values. This indicates that the manual annotations of these key points have a large spread and it is not easy even for humans to locate these key points consistently.

**Table 2:** 15 images were annotated by 10 users to obtain a covariance matrix per key point per image. The average covariance matrix for a single key point is obtained by finding the mean and standard deviation of the 15 covariance matrix

a	b
$\left (\begin {array}{cc} 9.06 \pm 6.37 & -0.127 \pm 2.64 \\ -0.127 \pm 2.64 & 6.20 \pm 3.99 \end {array}\right)$	$\left (\begin {array}{cc} 16.3 \pm 13.1 & 5.22 \pm 4.19 \\ 5.22 \pm 4.19 & 5.17 \pm 2.20 \end {array}\right)$
c	d
$\left (\begin {array}{cc} 12.1 \pm 14.2 & -2.50 \pm 3.73 \\ -2.50 \pm 3.73 & 2.75 \pm 2.07 \end {array}\right)$	${\left (\begin {array}{cc} 2.47 \pm 1.33 & -0.0481 \pm 0.584 \\ -0.0481 \pm 0.584 & 1.40 \pm 0.836 \end {array}\right)}$
e	f
$\left (\begin {array}{cc} 1.99 \pm 0.935 & 0.0319 \pm 0.655 \\ 0.0319 \pm 0.655 & 2.07 \pm 0.927 \end {array}\right)$	${\left (\begin {array}{cc} 1.72 \pm 1.17 & -0.0474 \pm 0.452 \\ -0.0474 \pm 0.452 & 1.38 \pm 0.652 \end {array}\right)}$
g	h
$\left (\begin {array}{cc} 2.35 \pm 1.48 & 0.263 \pm 0.840 \\ 0.263 \pm 0.840 & 2.21 \pm 1.62 \end {array}\right)$	$\left (\begin {array}{cc} 2.43 \pm 1.32 & 0.259 \pm 0.980 \\ 0.259 \pm 0.980 & 2.06 \pm 1.45 \end {array}\right)$
i	j
$\left (\begin {array}{cc} 2.22 \pm 1.32 & -0.141 \pm 0.710 \\ -0.141 \pm 0.710 & 1.35 \pm 0.633 \end {array}\right)$	$\left (\begin {array}{cc} 2.41 \pm 0.848 & 0.190 \pm 0.691 \\ 0.190 \pm 0.691 & 1.81 \pm 0.669 \end {array}\right)$
k	l
$\left (\begin {array}{cc} 12.3 \pm 9.36 & 6.16 \pm 6.01 \\ 6.16 \pm 6.01 & 8.91 \pm 5.29 \end {array}\right)$	$\left (\begin {array}{cc} 5.21 \pm 2.62 & -1.43 \pm 1.26 \\ -1.43 \pm 1.26 & 1.97 \pm 0.746 \end {array}\right)$
m	
$\left (\begin {array}{cc} 13.5 \pm 15.0 & -3.64 \pm 5.56 \\ -3.64 \pm 5.56 & 9.09 \pm 5.76 \end {array}\right)$	

## Results

### Key point detection results

**Fig. 8:**
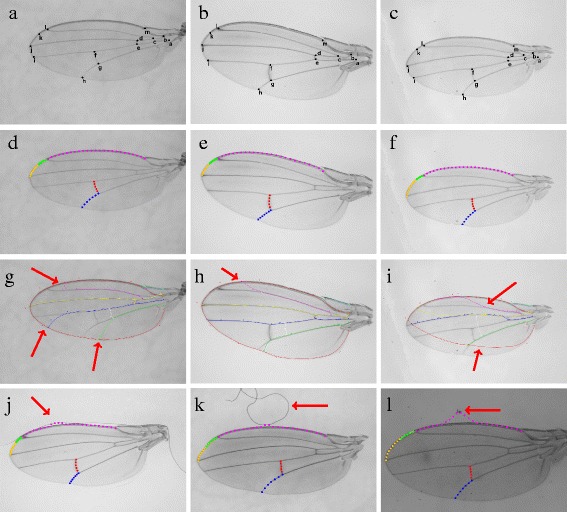
**a** - **c** shows some key point prediction results of our method. **d** -**f** shows arc lengths found using our method. The colours *red, blue, orange, green* and *magenta* represents the arc segments fg, gh, jl, kl, lm respectively. **g** - **i** shows key point and arc length predicted for the same images using the WINGMACHINE software. Red arrows are used to indicate the errors for clarity. The errors in **g** and **i** are due to incorrect prediction of joint locations while the error in **h** is due to a bad fitting results of an a priori template wing. Lastly, **j** - **l** shows three examples of bad arc length estimation results found using Active Contour due to artefacts in the background such as air bubbles and dust particles. The *red* arrows show the artefacts that affect the Active contour algorithm. There are a total of four images with bad arc length estimation out of our dataset of 600 images

Some results of Key Point Detection are shown in Fig. 8. To determine if a predicted key point is accurate, we check if the euclidean distance (in pixels) between the predicted key point and the ground truth is less than some threshold. The threshold used in our experiment is 2 standard deviations away from the corresponding key point’s covariance matrix. The results of this experiment are summarized in Table 3. The mean deviation in pixels for each point is also reported. Accuracy for key points ‘a’, ‘h’ and ‘k’ are not reported as these points were marked by the user. The mean pixel deviation is also slightly higher for key points ‘b’, ‘c’, ‘l’ and m’. These key points are located on slightly thicker fly wing veins as compared to other key points. A thicker vein would increase the candidate area for a key point and this explains the larger values in pixel deviations.

**Table 3:** Results obtained using images with the original resolution

	Dataset = 599				Dataset = 100			
	1 degree rotation		5 degree rotation		1 degree rotation		5 degree rotation	
Point	Within 2 stddev (%)	Mean Pixel Dev	Within 2 stddev (%)	Mean Pixel Dev	Within 2 stddev (%)	Mean Pixel Dev	Within 2 stddev (%)	Mean Pixel Dev
b	99.3	3.13±2.24	99.3	3.21±2.24	98.8	3.39±2.36	98.9	3.38±2.39
c	86.0	4.24±3.84	86.0	4.35±4.32	83.5	4.57±4.22	82.8	4.74±4.64
d	91.5	2.59±2.02	90.3	2.61±2.04	87.8	2.69±2.36	87.7	2.69±2.41
e	92.7	2.49±1.19	91.5	2.52±1.23	90.1	2.70±2.87	89.7	2.66±2.28
f	83.7	2.56±1.12	82.8	2.61±2.34	78.9	2.74±2.40	78.9	2.78±2.50
g	95.5	2.54±1.72	95.8	2.53±1.67	93.0	2.65±2.08	92.4	2.71±2.49
i	85.0	2.72±1.60	83.8	2.77±1.90	79.9	2.98±2.17	79.4	2.95±2.19
j	90.5	2.81±4.25	90.7	2.86±4.24	87.7	2.97±4.45	87.2	3.01±4.40
l	74.0	5.45±12.3	73.5	6.40±15.3	65.4	11.0±22.5	64.6	12.1±24.6
m	94.5	5.25±5.48	93.3	5.66±6.62	91.0	6.37±7.56	89.4	6.53±7.58

The results of using different a dataset of size 599 and 100 are shown as well. As shown in the table, using a larger dataset yields a more accurate result. Also note that key points a, h, k are excluded as these key points are annotated by the users and are thus not being predicted

Different sized data sets were used to predict key points. For the data set of size 100, 100 images had key points manually annotated and used for template matching. The remaining 500 images were used to test the accuracy of the algorithm. Cross validations was also done for this data set. Similarly, for the data set of size 599, 599 images had key points manually annotated and the remaining image was used for testing the algorithm. We expect that the data set of size 599 will yield better results. The accuracy results are shown in Table 3. We also downsized our images further and repeated the above experiments. The results of the downsized dataset can be found in Table 4.

**Table 4:** Results obtained using images 4 times smaller than the original resolution

	Dataset = 599				Dataset = 100			
	1 degree rotation		5 degree rotation		1 degree rotation		5 degree rotation	
Point	Within 2 stddev (%)	Mean Pixel Dev	Within 2 stddev (%)	Mean Pixel Dev	Within 2 stddev (%)	Mean Pixel Dev	Within 2 stddev (%)	Mean Pixel Dev
b	84.8	5.55±3.53	84.2	5.65±3.84	85.1	5.67±3.91	85.0	5.72±3.74
c	52.7	7.92±5.94	53.0	7.97±6.00	48.9	8.47±6.33	49.0	8.45±6.45
d	25.0	7.03±9.16	27.2	6.99±9.62	24.6	7.97±10.9	26.5	7.77±10.6
e	59.7	5.31±6.19	62.3	5.55±7.23	55.7	6.61±8.76	55.4	6.91±9.54
f	8.33	10.4±14.4	8.17	9.84±13.5	9.89	12.2±16.7	10.1	12.6±16.8
g	36.8	8.65±9.95	37.7	9.23±10.7	33.5	11.7±13.5	32.8	12.0±13.4
i	27.7	6.25±6.73	28.7	6.24±6.93	27.9	6.64±7.49	27.3	6.57±7.33
j	36.3	4.63±5.21	34.0	4.96±6.34	34.2	5.35±6.11	33.1	5.76±7.31
l	22.8	26.7±34.2	23.0	26.3±34.2	17.9	30.5±35.0	17.4	30.6±35.4
m	48.8	16.2±13.7	47.0	16.3±13.4	48.1	16.1±13.8	47.6	16.1±13.6

The results of using different a dataset of size 599 and 100 are shown as well. As shown in the table, using a larger dataset yields a more accurate result. Also note that key points a, h, k are excluded as these key points are annotated by the users and are thus not being predicted

We also compared our method against an existing fly wing fitting software, WINGMACHINE [22]. WINGMACHINE first detects curves in a fly wing image that fit a priori template before fitting spline curves to the image. The location of two landmarks - the humeral break and alula notch, need to be known before curve fitting can take place. We ran the Wings software on ten images in our dataset. The results are shown in Fig. 8 g – i and Table 5. WINGMACHINE tries to find the fly wing veins using edge detection methods and results were not so ideal in cases where there were background artefacts. Moreover, the edge detection algorithm sometimes fail to pick up some veins that are too faint. This can lead to a wrong prediction on the joint locations which results in a bad fit for the spline curves.

**Table 5:** A total of 10 fly wings images were used for prediction and the performance and accuracy of our method and WINGMACHINE is shown in this table. WINGMACHINE performs poorly in some cases when the wing veins or joints are faint and are not found using edge detection methods

Point	Accuracy of our method (%)	Mean pixel deviation	Accuracy of WINGMACHINE software method (%)	Mean pixel deviation
a	Marked by human	-	0	27.2±10.4
b	100.	2.59±1.36	60.0	14.6±16.4
c	100.	2.86±1.97	30.0	15.8±11.2
d	90.0	2.52±1.08	10.0	26.1±27.3
e	90.0	2.64±1.36	40.0	19.0±20.3
f	90.0	3.00±1.07	0	63.7±49.0
g	90.0	2.97±1.32	20.0	81.8±53.9
h	Marked by human	-	10.0	84.9±100.
i	60.0	2.86±1.84	50.0	75.8±104.
j	90.0	2.43±1.41	50.0	78.1±108.
k	Marked by human	-	N.A.	-
l	70.0	10.3±17.6	0	105.±106.
m	100.	3.27±2.69	60.0	24.9±37.8

### Arc length results

Some examples of good results of Active Contour are shown in Fig. 8. Note that even though there are dark patches (Fig. 8 d), background artefacts (Fig. 8 e) or spots on the wing (Fig. 8 f), Active Contour is still able to find the fly wing vein accurately most of the time.

We found a total of 4 bad results out of 600 images in our dataset. Users are allowed to edit bad results of arc lengths in the fly wing software. Some examples of bad results of Active Contour are also displayed in Fig. 8. As can been seen in Fig. 8 j and k, air bubbles are present in the background while dust is present in Fig. 8 l. It can be observed that only the location of arc lm is incorrect and that artefacts in these images lie above arc lm. It has been mentioned previously that the arc lm is initialized above the fly wing. These artefacts in the background produces large peaks in the active contour gradient image, resulting in Active Contour getting stuck in the local maxima of the artefact. Other arcs do not suffer from this problem as these arcs are initialized onto the foreground which has little artefacts. Thus only artefacts that lie just above the fly wing affects the accuracy of Active Contour.

The accuracy of our Active Contour method was also tested. Arc lengths ground truth of fifteen different fly wings, one for each species, are annotated manually and compared with the arc lengths found using Active Contour. The error is calculated as the area bounded by the annotated arc and the predicted arc. The error is then divided by the length of the annotated arc. The results are tabulated in Table 6. This is normal as a longer arc spans a larger area of the image and this increases the amount of noise and background artefact that might affect the Active Contour algorithm.

**Table 6:** Active Contour error measurement on 15 fly wings, one for each specie. Images are selected are random. The error is calculated as the area bounded by the annotated arc and the predicted arc. The error is then divided by the length of the annotated arc

Fly	ARC_F_G	ARC_G_H	ARC_J_L	ARC_K_L	ARC_L_M
D.bizonata f_1	0.495	0	0.383	1.061	3.077
D. sternopleuralis m_2	0.831	0.07	0.838	0.624	3.491
D.annulipes_m_13	0	0.111	0.12	1.44	6.658
D.buskii_f_20	0.583	0	1.602	0.658	6.776
D.coracina_m_10	1.124	0.383	1.712	1.761	3.975
D.curviceps f_2	0.263	0.012	0.734	1.645	5.795
D.hydei_m_14	0.446	0	0.562	1.521	6.948
D.immigrans f_18	0.05	0.133	0.546	0	6.229
D.lutescens_m_10	0.539	0.086	0.527	0.328	4.607
rufa_m_20	0.153	0.021	0.985	0.712	4.8
D. suzukii_m_03	0.709	0	0.202	0.14	4.254
Di.acutissima_m_17	0.954	0	0.826	1.741	3.99
H.sexbittata_f_22	0.734	0.237	1.034	2.289	4.048
L.area_m_13	1.828	0.093	0.156	0.846	2.833
S.graminum_f_17	0.637	0.019	1.19	2.476	4.499

### Species identification results

The efficiency of the wing image analysis for species identification is shown in Table 7. Out of 370 flies examined, 346 flies were identified as the correct species (success rate was 94%), whereas the capability of identifying correct sex was poor (success rate was 56%). This suggests that the wing morphology is not significantly different between female and male, whereas the between-species difference is significantly larger than the within-species difference among all of the 15 species examined. In addition, it is noteworthy that the present system perfectly recognized *Microdrosophila sp.*, which was not included in the training data, as ‘unknown’ species. This suggests that the present system has a discrimination power to give an appropriate answer even when the species of sample is not included in the training data. Therefore, the present system is expected to be useful to analyze wing morphology for species identification among species belonging to Drosophila and related genera, even after more data for more species are added.

**Table 7:** Efficiency of species identification using the wing image analysis

Species	No of samples	No of success for species	(%)	No of success for species and sex	(%)
*Dichaetophora acutissima*	3	3	100	-	-
*Drosophila annulipes*	14	14	100	7	50
*Drosophila bizonata*	18	16	89	10	56
*Drosophila busckii*	32	31	97	19	59
*Drosophila curviceps*	10	10	100	5	50
*Drosophila hydei*	17	15	88	6	35
*Drosophila immigrans*	38	33	87	29	76
*Drosophila lutescens*	20	20	100	-	-
*Drosophila rufa*	38	35	92	20	53
*Drosophila sternopleuralis*	38	36	95	17	45
*Hirtodrosophila sexvittata*	38	32	84	22	58
*Liodrosophila aerea*	38	38	100	25	66
*Scaptodrosophila coracina*	17	16	94	11	65
*Scaptomyza graminum*	24	22	92	8	33
*Microdrosophila sp.*	25	25	100	-	-
Total	370	346	94	179	56

Hyphens indicate that the sex-separated databases are not available. *Microdrosophila sp.* is not included in the training data and the success is defined as the case of identified as ’unknown’

## Discussion

Our software has managed to successfully predict landmarks on up to 15 different species of fly wing images. Incorrect landmarks detected can be corrected using the software user interface with ease. In addition, our software is able to accurately calculate and measure specific vein segments which are required for fly specie detection. Incorrect vein segments found can also be corrected using the user interface of the software.

In contrast to WINGMACHINE software, which makes use of high level features to optimize fit of an a priori model of a fly wing, our software utilizes low level features such as edges and pixel values to find land marks and vein segments. As mentioned by WINGMACHINE, developing detection algorithms using low levels features tend to encounter problems such as varying thickness of veins, uneven lighting and background artefacts which cause inaccuracies during prediction. However, our software manages to overcome the majority of these difficulties and performs well for images in our dataset.

One advantage of using WINGMACHINE over our software is that only two land marks are needed for detection instead of three. However, it should be noted that WINGMACHINE software may require pretreatment of image data and this may require more manpower as compared to our software. The advantage of using MorphoJ is that phenotypic data can be easily read and analysed within the software. However, MorphoJ does not have the capabilities to detect and extract these information from images automatically.

There are a few drawbacks for our algorithm. The first is the need for a large dataset. As demonstrated in the key point detection results section, a large dataset containing fly wing images of numerous fly species would help increase the accuracy of our algorithm. This results in a slow detection process as each image in the dataset has to be loaded. To reduce the time taken for the detection process, image patches centered on the fly wing’s key points are cropped from the original fly wing image and saved in a separate image. This separate image is loaded instead of the whole fly wing image. As the separate image is significantly smaller in size than the original fly wing image, loading it is much faster and thus reduces the time taken for key point detection. Although this method has worked well for our dataset, it is not an efficient solution where the size of the dataset can be very large.

The second disadvantage lies with our active contour algorithm. The veins that are currently being measured by active contour tend to lie on the circumference of the wing or on areas where there are no other veins lying close by. Our active contour may face problems measuring veins which have other separate veins in the surrounding area. In our active contour algorithm, we first initialize the vein segment as a straight line. In cases where there are no other veins that lie near the targeted vein, the vein segment will always evolve to the targeted vein. However, if there exist other nearby veins that run parallel to the targeted vein, active contour may choose to evolve the vein segment towards the other veins which are beside the target vein. Thus an improvement of the current active contour algorithm is required if it is needed to measure such veins in the future.

## Conclusion

In this paper, we have demonstrated how to locate *Drosophila* wing key points and obtain arc lengths of the wing veins using image processing methods. Our results show that our method is accurate and we compared our method against WINGMACHINE, another existing fly wing prediction software.

Listed here are some possible areas for future work. The first is to enhance our algorithm to perform key point predictions without the help of user defined key points. We could also improve the arc length finding method such that it is able to find the vein accurately even with background artefacts. Another possible area for future work would be to perform further research and analysis of the data extracted to build a fly species phenotypic tree. Lastly, our algorithm could be extended to predict more than just fifteen species of fly or perhaps be used to predict the species of other insects.

## Availability and requirements

**Project name:** DrosoWing

**Project home page:** http://evolgen.biol.se.tmu.ac.jp/fly/wing/

**Operating system:** Web browsers that support HTML5

**Programming language:** JavaScript, Java

**Requirements:** NA

**License:** NA

## Additional file

Additional file 1 Algorithms used in the paper. This supplementary document contains a detailed description of the algorithms used in this paper. The Key Point Detection algorithm is shown on page 1 while the Arc Length Detection algorithm can be found on page 2. (PDF 141 kb)

## Abbreviations

CLBP: Complete local binary pattern
GWAS: Genome wide association studies
HOG: Histogram of oriented gradient

## References

[1] Metzker ML (2010). Sequencing technologies the next generation. Nat Rev Genet.

[2] The 1000 G enomes Project Consortium (2010). A map of human genome variation from population-scale sequencing. Nature.

[3] Clark A (2007). Evolution of genes and genomes on the Drosophila phylogeny. Nature.

[4] Markow TA, O’Grady PM (2005). Drosophila: A Guide to Species Identification and Use.

[5] Silans LMN, Passerat De (1996). Wing morphometry of Phlebotomus perniciosus (Diptera: Psychodidae): calibration of methods with a laboratory population. Ann Trop Med Parasitol.

[6] Hall MJR, MacLeod N, Wardhana AH (2014). Use of wing morphometrics to identify populations of the Old World screwworm fly, Chrysomya bezziana (Diptera: Calliphoridae): A preliminary study of the utility of museum specimens. Acta Tropica.

[7] Francoy TM (2008). Identification of Africanized honey bees through wing morphometrics: two fast and efficient procedures. Apidologie.

[8] MORPHOMETRIC, COMPARATIVE (2005). Preliminary study of wing morphometry in relation to tsetse population genetics. Res Action.

[9] Rohlf FJ, Archie JW (1984). A comparison of Fourier methods for the description of wing shape in mosquitoes (Diptera: Culicidae). Syst Biol.

[10] Van Cann J (2015). Wing morphometrics as a possible tool for the diagnosis of the Ceratitis fasciventris, C. anonae, C. rosa complex (Diptera, Tephritidae). ZooKeys.

[11] Klingenberg CP, Leandro RM (2005). Distances and directions in multidimensional shape spaces: implications for morphometric applications. Syst Biol.

[12] Dickinson MH, Hannaford S, Palka J (1997). The evolution of insect wings and their sensory apparatus. Brain Behav Evol.

[13] Garcia-Bellido A, De Celis JF (1992). Developmental genetics of the venation pattern of Drosophila. Annu Rev Genet.

[14] Klingenberg CP, Zaklan SD (2000). Morphological integration between developmental compartments in the Drosophila wing. Evolution.

[15] Cowley DE, William RA, Rutledge JJ (1986). Quantitative genetics of Drosophila melanogaster. I. Sexual dimorphism in genetic parameters for wing traits. Genetics.

[16] Garcia-Bellido A (1975). Genetic control of wing disc development in, Drosophila. Cell patterning. Vol. 29.

[17] Diaz-Benjumea FJ, Cohen SM (1993). Interaction between dorsal and ventral cells in the imaginal disc directs wing development in Drosophila. Cell.

[18] Garcia-Bellido A, De Celis JF (1992). Developmental genetics of the venation pattern of Drosophila. Annu Rev Genet.

[19] Stark J (1999). The evolution and development of dipteran wing veins: a systematic approach. Annu Rev Entomology.

[20] Klingenberg CP, Zaklan SD (2000). Morphological integration between developmental compartments in the Drosophila wing. Evolution.

[21] Dedej S, Nazzi F (1994). Two distances of forewing venation as estimates of wing size. J Apic Res.

[22] Houle D (2003). Automated measurement of Drosophila wings. BMC Evol Biol.

[23] Klingenberg CP (2011). MorphoJ: an integrated software package for geometric morphometrics. Mol Ecol Resour.

[24] Zhou Y-H, Long-Bin L, James Rohlf F (1985). Automatic description of the venation of mosquito wings from digitized images. Syst Biol.

[25] Tofilski A (2004). DrawWing, a program for numerical description of insect wings. J Insect Sci.

[26] Schroder S (2002). The new key to bees: automated identification by image analysis of wings, Pollinating bees-the Conservation Link Between Agriculture and Nature..

[27] Weeks PJD (1999). Automating insect identification: exploring the limitations of a prototype system. J Appl Entomology.

[28] Crnojevic V (2014). Image processing method for automatic discrimination of hoverfly species. Math Probl Eng.

[29] Gibert P (2004). Comparative analysis of morphological traits among, Drosophila melanogaster and D. simulans: genetic variability, clines and phenotypic plasticity. Drosophila melanogaster, Drosophila simulans: So Similar, So Different.

[30] Sturtevant AH (1942). The classification of the genus Drosophila, with descriptions of nine new species, Vol. 4213.

[31] Wheeler MR, Takada H (1964). Diptera: Drosophilidae. Insects Micronesia.

[32] Mahalanobis PC (1936). On the generalized distance in statistics. Proc Nat Inst Sci India.

[33] De Maesschalck R (2000). The Mahalanobis distance. Chemometr Intell Lab Syst.

[34] R Core Team. R: A Language and Environment for Statistical Computing. Vienna: R Foundation for Statistical Computing; 2015. https://www.R-project.org/.

