# Supplementary material for: Semi-automated quantitative Drosophila wings measurements
Source: BMC Bioinformatics. 2017 Jun 28;18:319. doi: 10.1186/s12859-017-1720-y (PMC5490177; doi:10.1186/s12859-017-1720-y)
Supplement: Additional file 1 — Algorithms used in the paper. This supplementary document contains a detailed description of the algorithms used in this paper. The Key Point Detection algorithm is shown on page 1 while the Arc Length Detection algorithm can be found on page 2. (PDF 141 kb) [file 12859_2017_1720_MOESM1_ESM.pdf]

Algorithm 1 Key Point Detection Algorithm. **KEYPOINT-DETECTION**

procedure KEYPOINT-DETECTION(D, I) **Input:**

- D is the data set containing 600 annotated fly wing images from 16 *Drosophila* species. 13 key points are annotated per image and are labelled alphabetically from 'a' to 'm'.
- I is the test image where key points are to be found. The user is required to annotate 3 key points on the fly wing, namely 'a', 'h' and 'k', before key point detection can proceed.

Output

- The 13 predicted key points for test image I.

Function and variable description:

- TEMPLATE-MATCH($\tilde{w}^{D'_m}(x_j'^m, y_j'^m, \theta)$, $\tilde{w}^I(x_j'^m, y_j'^m, 0)$) $\tilde{w}^{D'_m}(x_j'^m, y_j'^m, \theta)$ and $\tilde{w}^I(x_j'^m, y_j'^m, 0)$ are the normalized image patches. Matching is done by sliding the window $\tilde{w}^{D'_m}(x_j'^m, y_j'^m, \theta)$ with respect to $\tilde{w}^I(x_j'^m, y_j'^m, 0)$. The pixel values have been normalized to values between 0 - 255. The output is a list of scores calculated as such:

$$s_{m,\theta}(c_x, c_y) = \|\tilde{w}^{D'_m}(x_j'^m, y_j'^m, \theta) - \tilde{w}^I(x_j'^m - c_x, y_j'^m - c_y, 0)\|$$

where c_x, c_y is the shift of the center locations between the image patches, with $-15 \leq c_x, c_y \leq 15$ and $\|\cdot\|$ is the Euclidean norm.

Start
for D_m in D **do**
  Obtain Q_M, the affine matrix, using key points 'a', 'h', 'k' of D_m and I
  Obtain D'_m, the transformed data set image, using Q_M
  for kp in 'a','b', ..., 'm' **do**
    Obtain transformed key point location $x_{kp}'^m, y_{kp}'^m$
    Obtain image patch $w^I(x_{kp}'^m, y_{kp}'^m, 0)$ from image I, centred at $x_{kp}'^m, y_{kp}'^m$.
    for θ in -10, -5, 0 ,5 , 10 **do**
      Obtain image patch $w^{D'_m}(x_j'^m, y_j'^m, \theta)$ from image D'_m, centred at $x_{kp}'^m, y_{kp}'^m$ and at the angle θ.
      $s_{m,\theta}^{kp}(c_x, c_y)$=TEMPLATE-MATCH($w^{D'_m}(x_j'^m, y_j'^m, \theta)$, $w^I(x_{kp}'^m, y_{kp}'^m, 0)$)
    end for
  end for
end for
for kp in 'a','b', ..., 'm' **do**
  Find the best match among all shifts, orientations and template images

$$D_m - (c_x^*, c_y^*, \theta^*, m^*) = \arg\min s_{m,\theta}^{kp}(c_x, c_y)$$

  Predicted key point kp coordinate is $(x_{kp}^I, y_{kp}^I) = (x_{kp}'^{m^*} - c_x^*, y_{kp}'^{m^*} - c_y^*)$
end for
return Predicted Key Points $(x_a^I, y_a^I), (x_b^I, y_b^I), ..., (x_m^I, y_m^I)$
end procedure

Algorithm 2 Arc Length Algorithm. **ARC-LENGTH**

procedure ARC-LENGTH(T, I) **Input:**

- T is the template wing that provides a good representation of wings for all different fly wings species. Arcs (FG, GH, JL, KL and LM) have been manually annotated on this template wing. Arcs will be transformed from the template wing to test image I for active contour.
- I is the test image where arc lengths are to be found.

Output

- The arc lengths of test image I.

Function and variable description:

- PREPROCESS(I) Preprocess the test image I for active contour. Returns the preprocessed image I_p.
- I_1 = Gaussian blur (I)
- I_2 = Invert(I_1)
- I_3 = Edge Detection(I_1)
- I_4 = Threshold(I_3)
- I_5 = Dilation(I_4)
- I_6 = Invert(I_5)
- I_7 = Distance Transform(I_6)
- I_8 = Invert(I_7)
- $I_p = I_2 + I_8$

I_p = PREPROCESS (I)

Obtain Q_T, the affine matrix, using key points 'a', 'h', 'k' of T and I

for arc in FG, GH, JL, KL, LM **do**

Obtain arc', the transformed arc, using Q_T

arc' can be represented as connecting straight line segments with n intermediate points $(x_1, y_1)...(x_{n-1}, y_{n-1})$

Define the objective function for arc'

$$L(x_1, y_1, ...x_{n-1}, y_{n-1}) = f(x_0, ...y_n, P) + \alpha \sum_{i=1}^{n} (l_i - l_0)^2$$

where

$$f(x_0, ...y_n, P) = \frac{\langle P \rangle}{2} \sum_{i=1}^{n} l_i \left[\frac{1}{P(x_{i-1}, y_{i-1}) + \epsilon} + \frac{1}{P(x_i, y_i) + \epsilon} \right]$$

and

$$\langle P \rangle = \frac{1}{2} \left[P(x_0, y_0) + P(x_n, y_n) \right]$$

and $\alpha \geq 0$ is a tuning parameter and $0 < \epsilon \ll 1$ is a small regularizer to prevent numerical overflow.

Minimize the objective function:

$$L(x_1^*, y_1^*, ...x_{n-1}^*, y_{n-1}^*) = \min_{x_1, ...y_{n-1}} L(x_1, ...y_{n-1})$$

Using gradient descend method,

$$(x_k^{(t+1)}, y_k^{(t+1)}) = (x_k^{(t)}, y_k^{(t)}) - \eta \partial_k L(x_1^{(t)}, ...y_{n-1}^{(t)})$$

The arc length arc' can then be calculated by:

$$I_{arc} = \sum_{i=1}^{n} \sqrt{(x_i^* - x_{i-1}^*)^2 + (y_i^* - y_{i-1}^*)^2}$$

end for

return Predicted Arcs $(I_{FG}), (I_{GH}), ..., (I_{LM})$

end procedure
